# Employees’ Perceptions of CSR, Work Engagement, and Organizational Citizenship Behavior: The Mediating Effects of Organizational Justice

**DOI:** 10.3390/ijerph16101731

**Published:** 2019-05-16

**Authors:** Tahir Farid, Sadaf Iqbal, Jianhong Ma, Sandra Castro-González, Amira Khattak, Muhammad Khalil Khan

**Affiliations:** 1 Department of Applied Psychology & Behavioral Science, Zhejiang University, 866 Yuhangtang Road, Hangzhou 310058, Zhejiang, China; tahir_khattak@zju.edu.cn (T.F.); sadaf@zju.edu.cn (S.I.); 2 Department of Business Organization and Commercialisation, Universidade de Santiago de Compostela, School of Business Administration, Lugo 27002, Spain; 3 Department of Marketing, Prince Sultan University, P.O. Box 66833, Riyadh 11586, Saudi Arabia; akhattak@psu.edu.sa; 4 College of Media and International Culture, Zhejiang University, 866 Yuhangtang Road, Hangzhou 310058, China; khan@zju.edu.cn

**Keywords:** employees’ perceptions, corporate social responsibility, distributive justice, procedural justice, organizational citizenship behavior, work engagement

## Abstract

Corporate social responsibility (CSR) at the individual level has emerged as an important field of research. However, a more comprehensive understanding of how CSR affects employee work engagement and organizational citizenship behavior (OCB) is still lacking. Based on social exchange theory, we examine the effects of employees’ perceptions of CSR on OCB and work engagement as well as the mediating mechanism of distributive and procedural justice, based on data collected from 350 employees working in the banking sector of Pakistan. Our study suggests that employees’ perceptions of CSR positively predict OCB and work engagement, and that work engagement is positively related to OCB. Both distributive and procedural justice positively mediate the effects of employees’ perceptions of CSR on OCB and work engagement.

## 1. Introduction

Corporate social responsibility (CSR) has been recognized as a key issue in business and in the academic literature of management, environment, and psychology [1,2,3]. CSR can be defined as “the policies and practices that mainly deal with an organization’s voluntary relationships with its community and societal stakeholders” [4]; it considered the firm’s socially responsible business strategies and practices that create macro- or organizational-level outcomes [5].

Empirical evidence has shown that CSR is important to internal stakeholders, particularly individuals working in organizations [1,2,3]. In this sense, there is evidence of the influence of CSR on job outcomes, such as job satisfaction [6,7,8], organizational commitment [1,9,10], turnover intention [11], organizational identification [12], organizational citizenship behavior [13,14], and work engagement [15]. However, despite the recent positive evidence of the impact of employees’ perceptions of CSR on a variety of job outcomes, studies on the influence of CSR on individual outcomes, such as attitudes and behaviors, have been largely absent [16], and there have been calls to fill this void in the literature.

First, even though several studies have examined the influence of CSR and organizational citizen behavior (OCB) of employees and of CSR and employee work engagement, both separately and jointly, there are still calls for more research into the influence of CSR on such attitudes and behaviors [11,15,17]. Thus, continuing to investigate these individual-level outcomes of CSR would be significant. Second, researchers have pushed for more studies to understand the mediating mechanisms underlying the relationship between CSR and employee outcomes [18]. Third, there is a need to examine the mechanism by which CSR can be linked with OCB and work engagement [19,20]. Fourth, very much related to the previous gap [19,20,21], there is a need to analyze the mechanism by which CSR can be linked with OCB and work engagement in greater depth. Fifth, considering the significant association between work engagement and OCB, a limited number of studies have been conducted on examining this association [22,23,24]. Finally, in line with those authors, we believe that there are gaps in the research related to the moral and psychological aspects that remain unresolved. Although several studies have recognized the connection between CSR and justice, only a limited number of studies have empirically tested it [25].

To fill these gaps, through the theoretical lens of social exchange theory [26] and the crucial role of organizational justice, which influences employee perception of job attitudes and behaviors [27], the current research examines the influence of employees’ perceptions of CSR related to OCB and work engagement through the mediation of distributive and procedural justice (a moral and psychological component). Rupp, Wright [28] argue that CSR and justice share the same structure of human needs. Therefore, it would be beneficial to understand how these two dissimilar organizational strategies with the same psychological background interact with each other. In addition, the study also examines the association between work engagement and OCB.

Finally, the current study examines the relationship in the context of developing economies, specifically Pakistan, and in the banking sector. This choice responds to the importance of the service industry in developing the economic life of a country [29], and its dynamic and useful role in the growth and strength of a developing country like Pakistan, where the banking sector is considered as a backbone of the national economy [30]. This contributes a new aspect to the existing literature, because previous studies investigated the employee perception of CSR and were predominantly conducted in the West or in the context of developed countries [2,13]. The banking sector of Pakistan provides a thought-provoking context for the analysis of these issues, because this sector has been studied from many different financial perspectives but, to the best of our knowledge, not from the perspective of the influence of socially responsible practices on employees. The banking sector plays a significant role in boosting the overall growth of a country’s economy. Empirical evidence reveals that due to the large amount of private banking in Pakistan, the sector is facing tough competition [31]. Further, due to the ongoing competition, most of the banks in this sector have expanded their boundaries by initiating Islamic banking and online banking services. In addition, past research findings reveal that when firms face such tough competition, they also seek supplementary marketing techniques in order to attain favorable customer attitudes and behaviors [32]. In order to achieve customer satisfaction and improve the company’s results, it is important to have employees who feel comfortable and committed and perform well for the betterment of the company. Thus, the present research is an attempt to examine employee’s perceptions of CSR and its influence on workplace behaviors. This implies that the results are relevant not only to Pakistan, but also to other countries with a similar socioeconomic and cultural context.

We observed a positive impact of CSR on employees, in that it encourages them to exhibit more cooperative behavior and show more engagement in their work. In addition, we also found that justice plays vital role in determining the relationship between CSR, OCB, and work engagement. This study makes a vital theoretical contribution to research on CSR, OCB, and work engagement by incorporating the mediating effects of distributive and procedural justice. The findings highlight the vital role of attitudinal variables (distributive and procedural justice) in affecting the relationship between CSR, OCB, and work engagement. The current study model also shows that employees’ perceptions of CSR affect their behavior, because pertinent CSR initiatives enlighten employees about organizational fairness, and thus, enhance their levels of OCB. From this perspective, we recommend that organizational management can play a vital role in nurturing the bond between the organization and its employees. In addition, firm management should provide shareholder-friendly governance to incentivize managers to engage in CSR-related activities.

The paper proceeds as follows. After this introduction, it develops the conceptual framework and the hypotheses. Then, in Section 3 and Section 4, it discusses the methodology and the results. Finally, it presents a discussion and conclusions.

## 2. Theory and Hypotheses

### 2.1. Social Exchange Theory

Social exchange theory (SET), presented by Blau [26], provides a strong theoretical framework for understanding employees’ attitudes and behaviors in an organization. As stated in [33], this theory is very important from the perspective of employee relations in the workplace. The authors of that study suggest that certain work antecedents imply interpersonal connections that normally lead to beneficial consequences for the organization for the development of positive attitudes or behaviors by employees. Other authors [34] have postulated four premises about this theory: every exchange interaction produces economic and/or social results; there is a process of comparison in time between the results obtained in such an exchange and those that could be obtained by other exchanges; if the results are positive over time, trust in the other party improves and the commitment to that exchange increases; and if the process is maintained over time, they end up creating rules of relational exchange that govern the relationship.

In sum, SET states that when a beneficial interaction of two parties occurs in a reciprocal relationship, the benefits do not have to be economic in either case, but may be determined by experience and the development of positive attitudes and behaviors between the two parties. According to this, at the workplace, for example, this theory posits interpersonal behaviors (bonds between the organization and supervisors or co-workers) in reciprocation of costs and rewards. If employees perceive that they are receiving benefits from the organization, they feel the need to give something back to the company.

### 2.2. Relationship between Employees’ Perceptions of CSR and OCB

OCB refers to an individual’s voluntary actions that are not formally recognized by the reward system but promote organizational effectiveness as a whole [35]. Past studies have suggested the roles of different organizational factors such as authentic leadership, organizational justice, and perceived organizational support that positively affect OCB among employees [36,37]. Researchers used to mostly focus on cognitive, effectual, and contextual factors [35]. Now, scholars have found a positive association of OCB with employee devotion and identification with an organization. OCB also assists in the operationalization of organizations and achieving goals [38,39,40].

Evidence reveals that at the workplace, individuals who recognize that the organization is socially responsible are more likely to demonstrate OCB [41,42]. A study conducted by Iqbal, Farid [14] showed a positive association between CSR and OCB among employees working in the banking sector of Pakistan. Abdullah and Rashid [13] found that CSR initiatives play a positive role in enhancing employees’ OCB. Gao and He [43] conducted a study of 220 employees working in different companies in China and found that CSR had a positive influence on OCB. Similarly, Rupp, Shao [2] conducted a study of 245 employees who were attending part time MBA classes at American university, and found that employees’ perception of external CSR has a positive influence on OCB. Lamm, Tosti-Kharas [44] found in their study that CSR has a positive influence on OCB. Overall, the empirical evidence reveals that CSR has a positive influence on OCB.

Social exchange theory presented in [26] let us know why employees exhibit extra-role behaviors. When employees perceive the firm’s CSR activities as fair, they exhibit cooperative behavior at the workplace in return. In addition, when organizations support their workers both socially and emotionally, the workers reciprocate in the form of appreciation [33]. The notion of reciprocity [45] indicates that workers feel obliged to compensate the favorable behaviors they experience in their firms. Based on social exchange theory and the above-mentioned empirical studies, this study hypothesizes the following:

**Hypothesis** **1:** Employees’ perceptions of CSR are positively associated with OCB.

### 2.3. Relationship between Employees’ Perceptions of CSR and Work Engagement

Work engagement is another attractive topic for researchers in the field of organizational behavior. It can be defined as a “positive, fulfilling work-related state of mind, categorized by vigor, dedication and absorption” [46]. Kahn [47] is considered the first scholar to initiate research on employees’ work engagement. Since then, a plethora of research has been conducted on work engagement [48]. Scholars have found personal resources and job resources as essential drivers of work engagement [49]. These resources enhance employees’ willingness to devote their efforts to work-related obligations and foster their personal growth, which positively influences their job outcomes, such as organizational commitment [50,51,52], individual performance [47], and job satisfaction [51,53], and ultimately enhances organizational performance [49,54].

On the basis of social exchange theory, we propose that the perception of CSR as a job resource positively influences employees’ work engagement. However, few studies have been conducted on employees’ perceptions of CSR and work engagement. As an exception, Gao, Zhang [15] showed a significant and positive association between CSR perception and employees’ work engagement. Similarly, another study showed a significant association between CSR perceptions and employees’ work engagement mediated by an organizational identification mechanism [55]. Glavas [17] conducted a study of 15,184 employees who were working in a large professional service in USA, and found that CSR has a positive and significant influence on employee work engagement. Moreover, Gao, Zhang [15] also found a positive link between CSR perception and work engagement mediated by perceived organizational support and Chinese values. Together, the above-mentioned studies indicate a positive association between CSR and work engagement.

Hence, we posit the following hypothesis:

**Hypothesis** **2**: Employees’ perceptions of CSR are positively associated with work engagement.

### 2.4. Relationship between Work Engagement and OCB

Based on the argument that psychological experience at work drives work behavior [47], we can say that OCB is closely related to commitment. Some authors [35] even consider that OCB is a type of behavioral commitment. In this sense, research has shown that work engagement positively influences in-role and extra-role behaviors, such as OCB [22,23,24]. Employees who are psychologically engaged in their work and with their company will be more likely to do things that their job position does not require and spend more time and effort on work-related issues and relationships, that is, OCB. On the basis of these previous arguments and the preceding empirical evidence, we propose the following hypothesis:

**Hypothesis** **3:** Employees’ work engagement is positively related to their OCB.

### 2.5. Mediating Role of Organizational Justice

Organizational justice is an interesting topic of research for scholars in the field of organizational psychology and management [56,57]. It refers to “employees’ perception of how fairly they are treated by the organization at the workplace” [58]. Distributive and procedural justice are the primary dimensions of organizational justice, and most empirical research has conceptualized these two constructs as perceived fairness. Early justice research focused on the distribution of outcomes [59,60]. Distributive justice can be defined as fairness in reward and resource distribution [60]. Workers consider rewards distribution to be fair if there is a balance between their contribution and the rewards [61]. Procedural justice refers to how employees perceive fairness in the process through which outcomes are reached and decisions made [62,63,64]. Procedural justice includes the extent to which a representative asks for and uses employee input, engages in two-way communication, gives employees the opportunity to challenge decisions, and constantly applies standard or rules [65,66].

We focus on distributive and procedural justice, since a meta-analytic review conducted by Colquitt, Conlon [36] revealed that they are highly linked with OCB and work engagement. Following the operationalization of Colquitt et al. (2001b), other studies have shown that employees´ perceptions of fairness are positively linked with both employee outcomes, OCB and work engagement [57].

When a company develops CSR practices, employees can feel that with these actions the company is distributing part of its resources, which in turn rewards their effort and dedication to the company. Rooted in social exchange theory (Blau, 1964), at the same time that employees feel they are receiving benefits from social responsibility actions, they feel an obligation to gratify the company. Thus, this argument suggests that employees will develop work engagement and extra-role behaviors such as OCB to give something back for what they receive.

**Figure 1:**
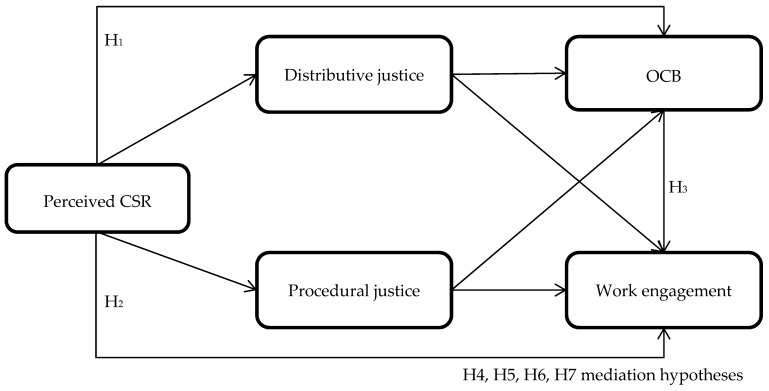
Proposed framework of the study. CSR, corporate social responsibility; OCB, organizational citizenship behavior.

Extending the previous reasoning, we argue that the moment employees perceive a fairness process when the company allocates CSR resources and actions, they will connect with the company. Again, based on the theory proposed by Blau (1964), employees will feel a need to give back to the company part of what they receive, and they will do so in the form of positive attitudes and behaviors. Hence, it is suggested that both distributive justice and procedural justice will positively mediates the association between CSR and OCB as well as between CSR and work engagement (Figure 1). We therefore hypothesize the following:

**Hypothesis** **4:** Distributive justice positively mediates the association between employees’ perceptions of CSR and OCB.

**Hypothesis** **5:** Procedural justice positively mediates the association between employees’ perceptions of CSR and OCB.

**Hypothesis** **6:** Distributive justice positively mediates the association between employees’ perceptions of CSR and work engagement.

**Hypothesis** **7:** Procedural justice positively mediates the association between employees’ perceptions of CSR and work engagement.

## 3. Research Method

### 3.1. Sample and Procedures

In this study, we used a cross-sectional design and collected data from 350 employees working in different private banking organizations in Peshawar city of Pakistan’s Khyber Pakhtunkhwa (KPK) Province. The use of this design is based on previous literature in the field of CSR [67,68,69], OCB [69,70,71], and work engagement [71,72,73].

The researchers visited the banks, highlighted the study’s significance to employees and persuaded them to participate in the study. Formal permission was obtained from every bank manager before collecting data from respondents. The researchers collected the data using self-administered questionnaires and assured the confidentiality of responses for respondents. The data were collected in February 2018 (one-month period). This study was carried out in accordance with the recommendations of the guidelines of the Ethics Commission of Zhejiang University with written informed consent from all participants. All of them gave written informed consent in accordance with the Declaration of Helsinki 1964. The study was approved by the Ethics Commission of the Department of Psychology and Behavioral Science, Zhejiang University.

By using convenient sampling, we distributed 400 questionnaires and received 350 completed questionnaires, representing a response rate of 87%. Among the respondents, 78% were female and 22% were male. A majority of respondents (63%) were 21–30 years of age, 30% were 31–40 years of age, and the remaining (7%) were 41–50 years of age. In addition, 51% of respondents were married. A majority of respondents (66%) held a master’s degree. Regarding work experience, 44% of respondents had 1–5 years’ work experience, 33% had 6–10 years’ work experience, 11% had 11–15 years’ work experience, 7% had 16–20 years’ work experience, and the remaining 5% had more than 20 years’ work experience. Finally, a majority of respondents (83%) were staff members, and the remaining (17%) were managers.

### 3.2. Measures

All responses were measured by using 5-point Likert-type scale [73], with anchors from “strongly disagree” to “strongly agree.” The original English version of the questionnaire was used for data collection. Upon first contact with small group of respondents, it was determined that they understood the questions adequately; therefore, a back-translation process was not necessary.

#### 3.2.1. Employees’ Perceptions of CSR

A 3-item scale adjusted from Hur, Kim [74] and Wagner, Lutz [75] was used to measure employees’ perception of CSR. The items were: “This organization is socially responsible,” “This organization is concerned with improving the well-being of society,” and “This organization behaves responsibly regarding the environment.” This scale has been used in several studies [12,76,77,78]. The reliability of the scale was 0.808.

#### 3.2.2. Distributive Justice and Procedural Justice

The scale developed by [79] was used to measure distributive and procedural justice. Distributive justice was covered by 5 items; for example: “I consider my workload to be quite fair” and “I think that my level of pay is fair.” The reliability of distributive justice was 0.860. Procedural justice was covered by 6 items; for example: “Staff members are allowed to change or appeal job decisions made by the management” and “My manager makes sure that all staff concerns are heard before job decisions are made.” The reliability of procedural justice was 0.881.

#### 3.2.3. Work Engagement

Employees’ work engagement was measured using the Utrecht Work Engagement Scale developed in [46]. The scale is composed of 9 items that include “I feel happy when I work intensely” and “I am proud of the work that I do.” The scale reliability was 0.908.

#### 3.2.4. Organizational Citizenship Behavior (OCB)

The scale developed in [80] was used to measured OCB. The scale comprises 8 items and includes “I show genuine concern and courtesy toward staff members, even under the most difficult business or personal situations” and “I willingly give my time to help other staff members who have work-related problems.” The reliability of the scale was 0.895.

#### 3.2.5. Control Variables

To avoid endogeneity issues, this study used education and organizational tenure as control variables and examined its association with work engagement and OCB. Past studies also controlled for the effect of education and organizational tenure and examined its influence on work engagement and OCB [81,82].

## 4. Results

### 4.1. Confirmatory Factor Analysis

To examine the construct validity, a set of confirmatory factor analyses (CFAs) were performed in AMOS (22). First, we conducted a baseline model (model 1, 5-factor) that was composed of all main variables—i.e., employees’ perception of CSR, distributive justice, procedural justice, OCB, and work engagement—to calculate the model fit indices (shown in Table 1) and compare with other models. The findings show a good model fit for the baseline model compared to other proposed models in the study: chi-square/degree of freedom (CMIN/DF) = 1.679, incremental fit index (IFI) = 0.952, comparative fit index (CFI) = 0.951, Tucker–Lewis index (TLI) = 0.946, root mean square error of approximation (RMSEA) = 0.044. Model 2 was a 4-factor model in which employees’ perception of CSR and distributive justice were combined into a new single factor. Model 3 was a 3-factor model in which employees’ perception of CSR, distributive justice, and procedural justice were combined into a single factor. Model 4 was a 2-factor model in which employees’ perception of CSR, distributive justice, and procedural justice were combined together and work engagement and OCB were merged into a new single factor. Model 5 was a 1-factor model in which employees’ perception of CSR, distributive and procedural justice, work engagement, and OCB were merged into a single factor to form a new bigger factor. CFA with maximum likelihood estimation was conducted for all 5 models. The factor loading for each factor was found to be significant, indicating good convergent validity. The average variance extracted (AVE) of all proposed variables was checked and the square root of every AVE was found to be greater than all the coefficients of the variables [83].

### 4.2. Descriptive Statistics

The means, standard deviations, and correlations of the variables are shown in Table 2. The results show that all variables are positively correlated in the direction as expected.

### 4.3. Regression Analysis Results

To test the main hypotheses, we conducted multiple linear regression. Table 3 and Table 4 indicate the effects of the independent variable (perception of CSR) and control variables (education and organizational tenure) on the dependent variables (OCB and work engagement) as well as the influence of work engagement on OCB. The findings in Table 3 indicate that employees’ perceptions of CSR are positively and significantly related to OCB (β = 0.363, *p* < 0.001), hence hypothesis 1 is supported. Similarly, hypothesis 2 predicts a positive association between employees’ perceptions of CSR and work engagement. Results indicate that employees’ perceptions of CSR have an important and positive relationship with work engagement (β = 0.410, *p* < 0.001), supporting hypothesis 2. Hypothesis 3 predicts a positive association between employees’ work engagement and OCB. Results indicate that employees’ work engagement has an important and positive relationship with OCB (β = 0.528, *p* < 0.0001), supporting hypothesis 3.

### 4.4. Mediation Analysis Results

In this study, Process for SPSS (version 22, IBM, Armonk, NY, USA) developed by [84] was used to analyze the mediation hypotheses. We chose model 4 to test the direct effect of employees’ perception of CSR on OCB and work engagement and the mediating effect of distributive and procedural justice. Further, 95% bias-corrected confidence interval with 5000 bootstrapping sample estimates was used.

In hypothesis 4, we assume that distributive justice will positively mediate the association between employees’ perceptions of CSR and OCB. The results presented in Table 5 show that employees’ perceptions of CSR have a significant positive relationship with distributive justice (β = 0.502, *p* < 0.0001), and distributive justice has a positive association with OCB (β = 0.221, *p* < 0.0001), supporting hypothesis 4. Similarly, hypothesis 5 predicts a positive mediating impact of procedural justice on the association between employees’ perceptions of CSR and OCB. The results, shown in Table 5, reveal that employees’ perceptions of CSR are positively linked with procedural justice (β = 0.426, *p* < 0.0001). Also, procedural justice has a positive link with OCB (β = 0.210, *p* < 0.0001), supporting hypothesis 5.

Hypothesis 6 predicts that distributive justice positively mediates the bond between employees’ perceptions of CSR and work engagement. The results in Table 6 indicate that employees’ perceptions of CSR are positively related to distributive justice (β = 0.502, *p* < 0.0001). In addition, distributive justice has a significant positive relationship with work engagement (β = 0.172, *p* < 0.0001), supporting hypothesis 6.

Likewise, hypothesis 7 predicts that procedural justice positively mediates the bond between employees’ perceptions of CSR and work engagement. The results in Table 6 indicate that employees’ perceptions of CSR are positively related to procedural justice (β = 0.426, *p* < 0.0001). There is also a significant positive relationship between procedural justice and work engagement (β = 0.258, *p* < 0.0001), meaning that hypothesis 7 is also supported.

## 5. Discussion

In the current study, we explored the positive effect of employees’ perceptions of CSR on OCB and work engagement and the positive mediating effect of distributive and procedural justice in the banking sector of Pakistan. We found a significant positive relationship between employees’ perceptions of CSR, OCB, and work engagement. We also found a mediating effect of distributive and procedural justice on this relationship.

This study broadens our understanding of employees’ perceptions of CSR and its theoretical underpinning by contributing to the CSR literature in the field of psychology in two prominent ways. First, past studies analyzed the effects of employees’ perceptions of CSR on different employee attitudes and workplace behaviors, such as organizational commitment [9,10], turnover intention [11], and organizational identification [12]. OCB and work engagement are important behavioral measures that play vital roles in boosting organizational performance [39,50]. Considering these vital roles, scholars have begun to show more interest in employees’ perceptions of CSR and its effects on OCB and work engagement [11,15,17,85]. We extended the studies concerning employees’ perception of CSR by exploring its effects on OCB and work engagement in Pakistan. Second, in line with past studies [23,24], we also found a positive association between work engagement and OCB. From the social exchange theory of Blau [26], we argue that when individuals perceive a firm’s CSR-related activities and policies as fair, they show more engagement in their work and are more likely to go beyond their formal duties by showing behaviors that benefit the overall firm. In addition, social exchange theory makes an important theoretical contribution to the literature in the field of psychology, as it is used extensively to clarify the studied association.

Third, identifying organizational justice dimensions (distributive and procedural justice) as an important underlying mechanism by linking employees’ perception of CSR with OCB and work engagement, this study responds to recent calls to examine the mediating mechanisms linking employees’ perceptions of CSR with individual-level job outcomes [19,20]. Our findings also provide empirical support for the role of organizational fairness in the perspective of CSR suggested by Rupp, Shao [2] and [7]. Additionally, these findings extend Jung and Ali [86] empirical results examining the mediating effect of distributive and procedural justice to highlight the connection between employees’ perceptions of CSR and job outcomes.

### 5.1. Theoretical Implications

The current study has several theoretical implications. It contributes significantly to the current literature [87,88] by introducing a way to cultivate new linkages between CSR, organizational justice, OCB, and work engagement in the context of the Pakistani banking sector. The findings reveal that CSR positively influences employees’ workplace behaviors (work engagement and OCB). This study also makes a theoretical contribution regarding CSR, OCB, and work engagement by incorporating the mediating effects of distributive and procedural justice. The findings highlight the vital role of attitudinal variables (distributive and procedural justice) in affecting the relationship between CSR, OCB, and work engagement. Last but not least, the study reveals the significant role of social exchange theory in explaining the relationship between dispositional variables with attitudinal and behavioral variables in Pakistan.

### 5.2. Practical Implications

This study also has several practical implications. First, we explored the positive impact of employees’ perception of CSR in terms of encouraging them to exhibit more cooperative behavior and show more engagement in their work. As workers are the prominent internal stakeholders [89], firms must pay more attention to their internal CSR-related activities. In addition, CSR activities directly influence employees, and they reciprocate with positive behavior. Hence, the top management must conduct its affairs with employees fairly to achieve sustainable development. The current study model also revealed that employees’ perception of CSR affects their behaviors because pertinent CSR initiatives enlighten employees about organizational fairness and thus enhance their levels of OCB. From this perspective, we recommend that organizational management can play a vital role in nurturing the bond between the organization and employees. As the literature identifies CSR contract as an important variable to improve firm performance [90], we suggest that firms must focus on CSR contract. We also suggest that firms should provide incentives for CSR for managers, which can be vital in boosting firm performance. Finally, this study will help future researchers to advance the association between employees’ perception of CSR, organizational justice, OCB, and work engagement.

### 5.3. Limitations and Future Suggestions

This study is not without limitations. First, the data collection was limited to only one sector. Future studies should examine and extend to other industries. Second, for data collection, a cross-sectional study design was used, which makes it difficult to generalize the results. Future research could use a longitudinal study design to avoid the uncertainty of causal relationships. Third, this study was conducted in Pakistan. To increase the generalizability of the findings, studies should replicate this study model in other developing countries. Fourth, our study claims causal effects but is weak in addressing the endogeneity problems. Future researchers should focus on instrumental variables such as firm size when examining the said relationship. Finally, researchers should examine the effect of CSR on other organizational and individual variables such as employees’ intention to stay, work ethic, happiness, and organizational culture.

## 6. Conclusions

We accomplished our study objectives by researching the banking sector in Pakistan. We observed a positive impact of CSR in terms of encouraging employees to exhibit more cooperative behavior and show more engagement at the workplace. We also found a positive association between work engagement and OCB. In addition, we found that justice plays a vital role in determining the relationship between CSR, OCB, and work engagement. The current paper makes an important contribution to the existing literature in the field of CSR perceptions and related constructs, namely justice, work engagement, and OCB. By using social exchange theory, the study also makes a significant contribution to the existing literature to explain the studied variables. This study also has practical significance, as it addresses how employees’ perceptions of CSR influence their behaviors and how they reciprocate by showing more engagement in their work and exhibiting more citizenship behavior in terms of helping their colleagues and supervisors. The study suggests that top management reconsider the role of CSR initiatives by clearly focusing on justice to boost employees’ OCB and work engagement. This study also explores a more inclusive view of how CSR initiatives can be used to improve employees’ job outcomes in the banking sector. Future research is encouraged to conduct more studies on CSR-related issues from the employee perspective in diverse cultures of developing and underdeveloped countries.

## Figures and Tables

**Table 1:** Results of confirmatory factor analysis.

Measurement Model	CMIN/DF	IFI	CFI	TLI	RMSEA
M1: 5-factor	1.679	0.952	0.951	0.946	0.044
M2: 4-factor	1.740	0.947	0.947	0.941	0.046
M3: 3-factor	1.731	0.950	0.949	0.942	0.046
M4: 2-factor	2.113	0.926	0.925	0.911	0.056
M5: 1-factor	2.345	0.912	0.911	0.893	0.062

CMIN/DF, chi-square/degree of freedom; IFI, incremental fit index; CFI, comparative fit index; TLI, Tucker-Lewis index; RMSEA, root mean square error of approximation.

**Table 2:** Means standard deviation (SD) and correlation.

Variable	Mean	SD	1	2	3	4	5
Perception of CSR	4.23	0.771	1				
Distributive justice	3.91	0.939	0.413 **	1			
Procedural justice	4.08	0.776	0.424 **	0.352 **	1		
Work engagement	4.07	0.845	0.374 **	0.313 **	0.353 **	1	
OCB	4.04	0.836	0.335 **	0.344 **	0.302 **	0.528 **	1

*N* = 350; ** *p* < 0.01 (2-tailed), CSR: corporate social responsibility, OCB: organizational citizenship behavior.

**Table 3:** Regression analysis results of perception of CSR, OCB, and work engagement (WE).

Variable	OCB	WE
Control		
Education	−0.065	−0.154 **
Organizational tenure	0.074	0.014
Perception of CSR	0.335 ***	0.374 ***
R^2^	0.112	0.140
Adjusted R^2^	0.109	0.138
F	43.851 ***	56.709 ***

*** *p* < 0.0001, ** *p* < 0.01.

**Table 4:** Regression analysis results of work engagement and OCB.

Variable	OCB
Education	−0.065
Organizational tenure	0.074
Work engagement	0.528 ***
R^2^	0.279
Adjusted R^2^	0.276
F	107.494 ***

*** *p* < 0.0001.

**Table 5:** Coefficients for mediating effects.

Testing Path	Hypothesis 4						Hypothesis 5					
	Unstandardized Coefficient		*t*	Sig	Bootstrapping		Unstandardized Coefficient		*t*	Sig	Bootstrapping	
	Coeff.	SE			LLCI	ULCI	Coeff.	SE			LLCI	ULCI
IV → MV (a)	0.502	0.059	8.456	0.001	0.386	0.619	0.426	0.049	8.722	0.001	0.330	0.522
MV → DV (b)	0.221	0.048	4.592	0.001	0.126	0.315	0.210	0.059	3.553	0.001	0.094	0.326
IV → MV → DV (c׳)	0.252	0.058	4.307	0.001	0.137	0.367	0.273	0.059	4.593	0.001	0.156	0.390
IV → DV (c)	0.363	0.055	6.622	0.001	0.255	0.470	0.363	0.055	6.622	0.001	0.255	0.470
Indirect effects	0.111	0.031			0.058	0.182	0.089	0.032			0.036	0.165

Hypothesis 4: IV (perception of CSR), MV (distributive justice), DV (OCB); hypothesis 5: IV (perception of CSR), MV (procedural justice), DV (OCB), LLCI (lower level confidence interval), ULCI (upper level confidence interval).

**Table 6:** Coefficients for mediating effects.

Testing Path	Hypothesis 6						Hypothesis 7					
	Unstandardized Coefficient		*t*	Sig	Bootstrapping		Unstandardized Coefficient		*t*	Sig	Bootstrapping	
	Coeff.	SE			LLCI	ULCI	Coeff.	SE			LLCI	ULCI
IV → MV (a)	0.502	0.509	8.456	0.001	0.386	0.619	0.426	0.049	8.722	0.001	0.330	0.522
MV → DV (b)	0.172	0.048	3.551	0.001	0.077	0.267	0.258	0.058	4.431	0.001	0.143	0.372
IV → MV → DV (c׳)	0.324	0.059	5.506	0.001	0.208	0.439	0.300	0.059	5.125	0.001	0.185	0.415
IV → DV (c)	0.410	0.054	7.531	0.001	0.303	0.517	0.410	0.054	7.531	0.001	0.303	0.517
Indirect effects	0.502	0.509	8.456	0.001	0.386	0.619	0.110	0.034			0.052	0.187

Hypothesis 6: IV (perception of CSR), MV (distributive justice), DV (work engagement); hypothesis 7: IV (perception of CSR), MV (procedural justice), DV (work engagement), LLCI (lower level confidence interval), ULCI (upper level confidence interval).
